# Effects of maternal nutrient restriction during the periconceptional period on placental development in the mouse

**DOI:** 10.1371/journal.pone.0244971

**Published:** 2021-01-14

**Authors:** Gerialisa Van Gronigen Case, Kathryn M. Storey, Lauren E. Parmeley, Laura C. Schulz

**Affiliations:** 1 Department of Obstetrics, Gynecology and Women’s Health, University of Missouri, Columbia, MO, United States of America; 2 Division of Biological Sciences, University of Missouri, Columbia, MO, United States of America; Xavier Bichat Medical School, INSERM-CNRS - Université Paris Diderot, FRANCE

## Abstract

Maternal undernutrition has detrimental effects on fetal development and adult health. Total caloric restriction during early pregnancy followed by adequate nutrition for the remainder of gestation, is particularly linked to cardiovascular and metabolic disease risks during adulthood. The placenta is responsible for transport of nutrients from the maternal to fetal circulation, and the efficiency with which it does so can be adjusted to the maternal nutrient supply. There is evidence that placental adaptations to nutrient restriction in early pregnancy may be retained even when adequate nutrition is restored later in pregnancy, leading to a potential mismatch between placental efficiency and maternal nutrient supplies. However, in the mouse, 50% caloric restriction from days 1.5–11.5 of gestation, while temporarily altering placental structure and gene expression, had no significant effect on day 18.5. The periconceptional period, during which oocyte maturation, fertilization, and preimplantation development occur may be especially critical in creating lasting impact on the placenta. Here, mice were subjected to 50% caloric restriction from 3 weeks prior to pregnancy through d11.5, and then placental structure, the expression of key nutrient transporters, and global DNA methylation levels were examined at gestation d18.5. Prior exposure to caloric restriction increased maternal blood space area, but decreased expression of the key System A amino acid transporter *Slc38a4* at d18.5. Neither placental and fetal weights, nor placental DNA methylation levels were affected. Thus, total caloric restriction beginning in the periconceptional period does have a lasting impact on placental development in the mouse, but without changing placental efficiency.

## Introduction

Chronic diseases are leading causes of morbidity and mortality. For example, in the United States more than 29.1 million people (9.3%) are diabetic, and at least one-third of these cases in adults and 17% in children are associated with obesity [1]. Heart disease is the leading cause of death, accounting for 1 in every 4 deaths in the U.S. [2]. Though genetics, improper diet and lack of exercise play roles in predisposing adults to these chronic diseases, a growing body of evidence links their incidence to prenatal, environmental insults. In particular, maternal nutrient restriction, whether in the form of total caloric restriction, protein deficiency, or micronutrient deficiencies, predisposes offspring to heart disease [3–5] and obesity [6] in adulthood in both humans and animal models. One interpretation of these findings is that organisms may undergo adaptive responses to their environment (e.g. nutritional scarcity) that may prove maladaptive when the environment changes, but can no longer be reversed once sensitive developmental windows have closed [7].

In the classic Dutch Hunger Winter Study, higher rates of obesity and cardiovascular disease were found in adult sons of mothers that received only about 50% of normal calories during the first trimester, followed by an adequate diet later in gestation [8, 9]. In contrast, those subjected to the famine in later gestation did not have elevated rates of these diseases, though they were more susceptible to airway disease, and diabetes risk was increased by exposure at any fetal stage [10, 11]. First trimester exposure to the Chinese Great Famine was also particularly highly associated with hypertension in adulthood [12]. In sheep, nutrient restriction during early-mid gestation likewise increases adiposity [13, 14] and cardiovascular dysfunction [15, 16], and alters hypothalamic-pituitary-adrenal axis function in offspring [17, 18].

The mechanisms by which the particular combination of early gestation undernutrition and adequate nutrition in mid-late gestation programs poor adult outcomes remain poorly understood. The placenta is the organ responsible for the transfer of nutrients from the mother to the fetus. It is also in direct contact with both the maternal and fetal circulations. The placenta therefore can respond to maternal environmental stimuli to in turn affect the growing fetus [19]. There is evidence that the placenta itself may adapt to undernutrition during early gestation, and in some cases if adequate nutrition is restored, it may even grow larger than normal. For example, women that were exposed to the Dutch Hunger Famine during the first trimester and received an adequate diet in later pregnancy delivered significantly larger placentas than control mothers [20]. Similarly, placentas of ewes born to mothers who were undernourished through mid-pregnancy were larger than normal at birth [21–23]. Interestingly, in these cases, offspring were normal weight at term, with greater adipose tissue in the lamb, suggesting that placental adaptations compensated or even overcompensated for early nutrient restriction [20, 23]. On the other hand, maternal food restriction prior to and during the first half of gestation in guinea pigs reduced placental surface area and labyrinth zone size [24]. In the mouse, 50% caloric restriction from days 1.5–11.5 of gestation initially reduced placental weight and the size of junctional zone, but after restoration of adequate nutrition from days 11.5–18.5, placental size and structure recovered [25, 26]. These findings suggest that differences in the timing of caloric restriction interact with species differences to determine long-term impacts on placental development.

Here, we test the hypothesis that caloric restriction will permanently impact the mouse placenta if it occurs over the periconceptional period. Periconception is defined as the period from oocyte maturation and selection through early embryo development [27]. Particularly critical events during this time include the establishment of parent-of-origin specific DNA methyation, the erasure of most DNA methylation in non-imprinted regions, the separation of embryonic and extraembryonic lineages, and the beginnings of lineage-specific DNA methylation [27, 28]. Multiple studies show that exposing gametes and embryos to an adverse environment in vitro, prior to implantation in a normal uterine environment, is sufficient to alter embryo development and adult health [29]. In vivo, feeding mice a low protein diet only during the periconceptional period led to smaller, more efficient placentas at d17 [30], and to greater glucose and System A amino acid transport activity on d19 [31]. However, the long-term placental effects of total caloric restriction during the periconceptional period, such as that experienced in the Dutch and Chinese famines, have not been examined. As we have previously demonstrated that placental effects of food restriction from days 1.5–11.5 are reversible [25], here, mice were subjected to 50% caloric restriction beginning 3 weeks prior to mating, when oocyte maturation begins. Mice were returned to unlimited access to control diet for the latter half of gestation (d11.5), and then placental structure, nutrient transporter expression and global DNA methylation levels were determined near term (d18.5).

## Materials and methods

### Animals

**Fig 1:**
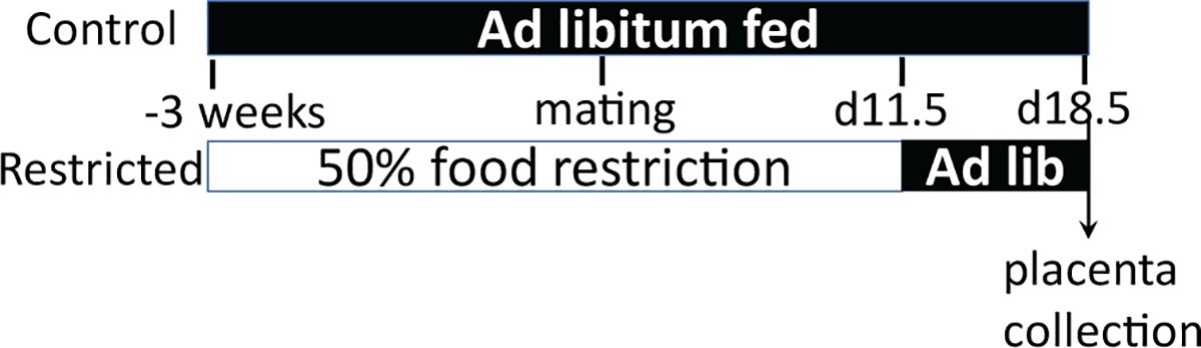
Experimental timeline. After one week of acclimation and measurement of consumption, female mice were randomized to *ad libitum* feeding (control), or fed 50% of previous consumption levels. Three weeks later, females were bred, and all were returned to *ad libitum* feeding at gestation d11.5 before tissue collection at d18.5.

All animal procedures were approved by University of Missouri Institutional Animal Care and Use Committee. Swiss Webster mice 6–8 weeks old and ≥ 25g were obtained from Envigo (Indianapolis, IN). They were individually housed in a 12hr dark, 12hr light cycle and fed an AIN-93G (Research Diets, Inc) growing rodent diet *ad libitum* for one week to acclimatize them to their surroundings. Specialized feeding containers were used so that daily food consumption could be recorded [32]. The experimental timeline is shown in **Fig 1**. Beginning three weeks prior to mating, female mice were randomly separated into two groups: a control group fed *ad libitum* and a restricted group fed 50% of previous average daily food consumption for each individual. Mice were fed at 2 pm each day. During the mating period, males were placed in the female cages each night at 8 pm and returned to their own cages each morning, with free access to food until a copulatory plug was detected for up to five nights. Females on the nutrient restricted diet consumed their entire food allotment each day before males were added to the cage. The day a copulatory plug was observed was termed d0.5 of gestation. Dietary treatments continued until d11.5, and then all mice were fed *ad libitum* from d11.5 to d18.5. All animals were allowed free access to water. At d18.5, mice were sacrificed, and placentae collected to examine placental morphology and gene expression. In total, tissues were collected from five mice in the control group and six in the restricted group.

### Tissue collection

At d18.5, pregnant females were euthanized by CO_2_ asphyxiation and cervical dislocation, and fetuses were euthanized by decapitation. Placentae were collected, and placental and fetal weights were recorded. One half of each placenta was fixed in 4% paraformaldehyde in PBS overnight for histological analysis. The second half of each placenta was divided into two pieces, one for gene expression analysis and the second for DNA methylation analysis. Both were snap frozen in liquid nitrogen and stored at -80°C until needed.

### Placental morphology

Fixed placentae were dehydrated and embedded in paraffin. Placentae were cut into 6 serial mid- sagittal sections, 5um thick, 50um apart, mounted on glass slides and stained with hematoxylin and eosin (H&E) (IDEXX Bioresearch, Columbia, MO). The largest cross-section on each slide was chosen for morphological analysis. For determination of total cross-sectional area and that of each of the major zones, overlapping images were photographed with a 4x objective lens on a Zeiss Axiovert 200M microscope, and arranged into a single, high-resolution image of the entire placental cross-section. Junctional zone and labyrinth zone areas were measured by manual outlining using the freehand selection tool in ImageJ software (NIH) by an operator blinded to treatment groups. Three placentas were chosen at random from each litter for the morphometric analysis by drawing three tubes from a bag.

To identify fetal blood vessels in the labyrinth zone, cross sections were immunohistochemically stained for PECAM1 (CD31) by using VECTASTAIN Elite ABC HRP Kit (Peroxidase, Rabbit IgG). Briefly, sections were rehydrated in a graded alcohol series, and antigen retrieval was performed by boiling in sodium citrate buffer (10mM Na Citrate, 0.05% Tween 20, pH 6.0) for 3 minutes in a pressure cooker, and then incubated in normal goat serum diluted according to manufacturer’s instructions to block non-specific binding. Placental sections were then incubated with anti-PECAM1 (Abcam ab28364) overnight at 4C. Secondary antibody and color development were carried out according to Vectastain instructions, and nuclei were counterstained with hematoxylin. Five representative images were photographed per placenta with a 40x objective on an Olympus IX81 microscope. The total cross-sectional areas of maternal blood sinuses and fetal blood vessels were determined by using the freehand selection tool within ImageJ software.

### Real time RT-PCR

Three placentas were chosen at random from each litter for analysis of gene expression. Placental tissue was homogenized in Trizol Reagent (Sigma Aldrich) with an Omni GLH Homogenizer. After phase separation, RNA was further purified using Qiagen RNeasy kit (QIAGEN, Valencia, CA) according to manufacturer’s protocol. RNA concentration was determined by absorbance using the Take3 Plate (Bio-Tek Instruments). Two μg of total RNA was reversed transcribed with SuperScript III First-Strand Synthesis System (Invitrogen/Life Technologies, Grand Island, NY), using random hexamer primers, according to manufacturer’s protocol.

Real-time RT-PCR was used to measure relative transcript levels of genes specific to the major trophoblast lineages: heart and neural crest derivative 1 (*Hand1*) was selected as a marker for trophoblast giant cells [33]; trophoblast specific protein alpha (*Tpbpa*) for spongiotrophoblast and glycogen cells [34, 35]; extraembryonic spermatogenesis homeobox 1 homolog (*Esx1*) for synctiotrophoblast cells [36]; and platelet and endothelial cell adhesion molecule 1 (*Pecam1*, CD31) for endothelial cells [37]. Placental lactogen 2 (*Prl3b1*) marks spongiotrophoblast, parietal and sinusoidal trophoblast giant cells, and has been found to be regulated by fasting at the protein, but not mRNA level [38–40]. Cathepsin Q (*Ctsq*) is a marker of sinusoidal trophoblast giant cells [38, 41].

Additionally, messenger RNAs encoding six key placental nutrient transporters were examined. The placenta expresses *Cd36* (FAT), a key fatty acid transporter, and *Srebp1c¸* (Sterol regulatory element binding transcription factor 1), a regulator of fatty acid synthesis [42, 43]. *Slc2a3* (GLUT3) is the major placental glucose transporter and has previously been shown to be regulated by maternal nutrition [44–46], as have the amino acid transporters *Slc7a5* (LAT1) and *Slc38a4* (SNAT4) [44, 47, 48].

Primers (**Table 1**) were synthesized by Integrated DNA Technologies. Primers for *Slc7a5* were predesigned by IDT (Mm.PT.58.10563809). The specificity and efficiency of each reaction was validated by using serial dilutions of placental cDNA. PCR was conducted in triplicate using RT^2^ SYBR Green ROX qPCR Master Mix (Qiagen) in a final volume of 12μl on a CFX Connect Thermocycler (Bio-Rad). Cycling conditions were as follows: 95°C for 10 minutes, 40 cycles of 95°C for 15 secs, 60°C for 1 minute. No-template and no reverse transcriptase controls were performed. Data from each gene of interest were normalized to the average values of housekeeping genes hypoxanthine phosphoribosyltransferase 1 (*Hprt*) and Glyceraldehyde-3-Phosphate Dehydrogenase (*Gapdh*), which were determined not to change with nutrient restriction. Fold change differences in gene expression and error bars were calculated using the ΔΔCt method [49].

**Table 1:** PCR primers.

Gene	Forward primer	Reverse primer	Source
*Cd36*	GGTCCTTACACTACAGAGTTCGTTA	CATTGGGCTGTACAAAAGACACA	[25]
*Ctsq*	CATTGCCAGTTGACAACACAAG	ATAGCCTTCATTTCGCCAATCA	[50]
*Esx1*	CCCATGCATCCTCAAATGATG	GCCTAAATGGTGGAGGCATTC	[51]
*Gapdh*	TGCACCACCAACTGCTTAGC	GGCATGGACTGTGGTCATGAG	[52]
*Hand1*	GAGATGTATACCTGAGAGCAACAGGCATGATAGGTAG	CTTCTCCTTCATTTCTTTCCTTTTCCTTC	[53]
*Hprt*	TGACACTGGCAAAACAATGCA	GGTCCTTTTCACCAGCAAGCT	[26]
*Prl3b1*	AGCAGCCTTCTGGTGTTGTC	TGTGACACCACAATCACACG	[54]
*Pecam1*	AGGCTTGCATAGAGCTCCAG	TTCTTGGTTTCCAGCTATGG	[55]
*Rbm31xy*	CACCTTAAGAACAAGCCAATACA	GGCTTGTCCTGAAAACATTTGG	[56]
*Slc2a3*	CTCTTCAGGTCACCCAACTACGT	CCGCGTCCTTGAAGATTCC	[57]
*Slc7a5*	GGCGTACATCAGCGTCAT	GTTCACGTCCTCAAGACTGTT	IDT
*Slc38a4*	TCACACTGCTGTTTCCAAGG	CAGCCGGAAGAATGAAAATC	[58]
*Srebp1c*	TGGTGGGCACTGAAGCAAA	GCAAGAAGCGGATGTAGTCGAT	[25]
*Tpbpa*	GCCAGTTGTTGATGACCCTGA	CCCATCGCCACTCTCTGTGT	[59]

### DNA isolation and methylation assay

Genomic DNA was isolated as previously described [60] from three placentas per dam, chosen by using a random number generator. Briefly, tissues were lysed in lysis buffer with proteinase K followed by separation using Phenol:Chloroform:Isoamyl Alcohol. Genomic DNA samples were stored at -20⁰C until use. DNA concentration was measured using an Epoch Microplate Spectrophotometer (Bio-Tek Instruments).

The level of global DNA methylation was measured using the 5-mC DNA ELISA kit (Zymo Research) according to the manufacturer's protocol, with the following exceptions: (1) Antibody incubation was performed overnight at 4⁰C; (2) 200ng of DNA was used per standard and sample well. To construct a standard curve, methylated DNA (Zymo Research) and unmethylated Lambda DNA (New England BioLabs) were mixed in various proportions (12.5%, 6.25%, 3.125%, 1.56%, 0.78%, and 0% methylated DNA). Intra-assay coefficient of variation was 5.41%. Samples were run in duplicate. To calculate the percentage of CpG that are methylated, the %5-methylcytosine (mC) calculated from the standard curve was corrected for the differences in average CpG density between mouse and *E*.*Coli* (used for the standard curve) genomic DNA, according to manufacturer’s instructions.

### Sex determination

RNA was isolated and reverse transcribed or DNA was isolated, as described above for each placenta used for the morphometry, RT-PCR, or methylation analyses. Sex was then determined by amplification of a 269 bp region of *Rbm31x* and the corresponding 353 bp region of *Rbm31y* by PCR (**Table 1**) [56].

### Statistical analysis

Fetal and placental weights were averaged for each litter, and these averages were compared between control and restricted groups by using Student’s t-test in GraphPad Prism software. Morphological features were analyzed by using the Mixed Procedure in SAS, with treatment group and placental sex as independent variables and litter nested within treatment group as a covariate, to account for the interdependence of placentas from the same litter. The same method was used to compare delta Ct values for analysis of real-time RT-PCR data. Data were termed statistically significant if p< 0.05.

## Results

### Placental and fetal growth

**Fig 2:**
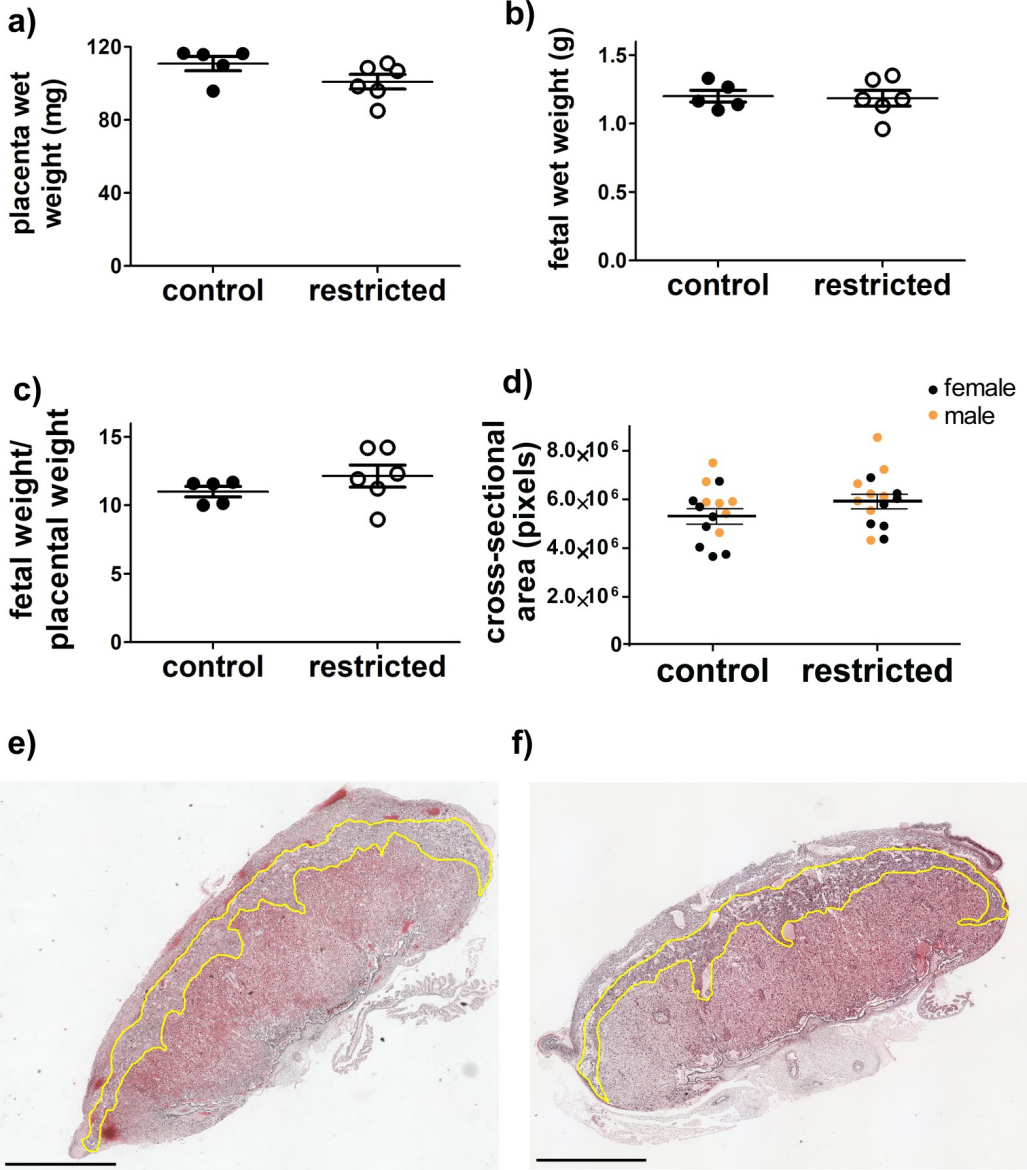
There were no significant differences between control fed and food restricted dams on d18.5 in **(a)** placental wet weights (**b)** fetal wet weights (**c)** placental efficiency (fetal weight/placental weight) or **d)** total cross-sectional area. **a-c)** Each marker represents the mean from one litter. Horizontal lines are the means for each maternal treatment group (control or restricted dams), and bars represent SEM. In **d)** each marker represents one placenta and lines represent least square means and SEM for each maternal treatment group. For a-d, N = 5 control, 6 restricted dams. Representative images of cross-sections of the placenta from **e)** control and **f**) food restricted. Junctional zones are outlined in yellow, with the decidua above and to the right, and labyrinth below left. Scale bar represents 1 mm. No grossly abnormal pathology was observed.

**Fig 3:**
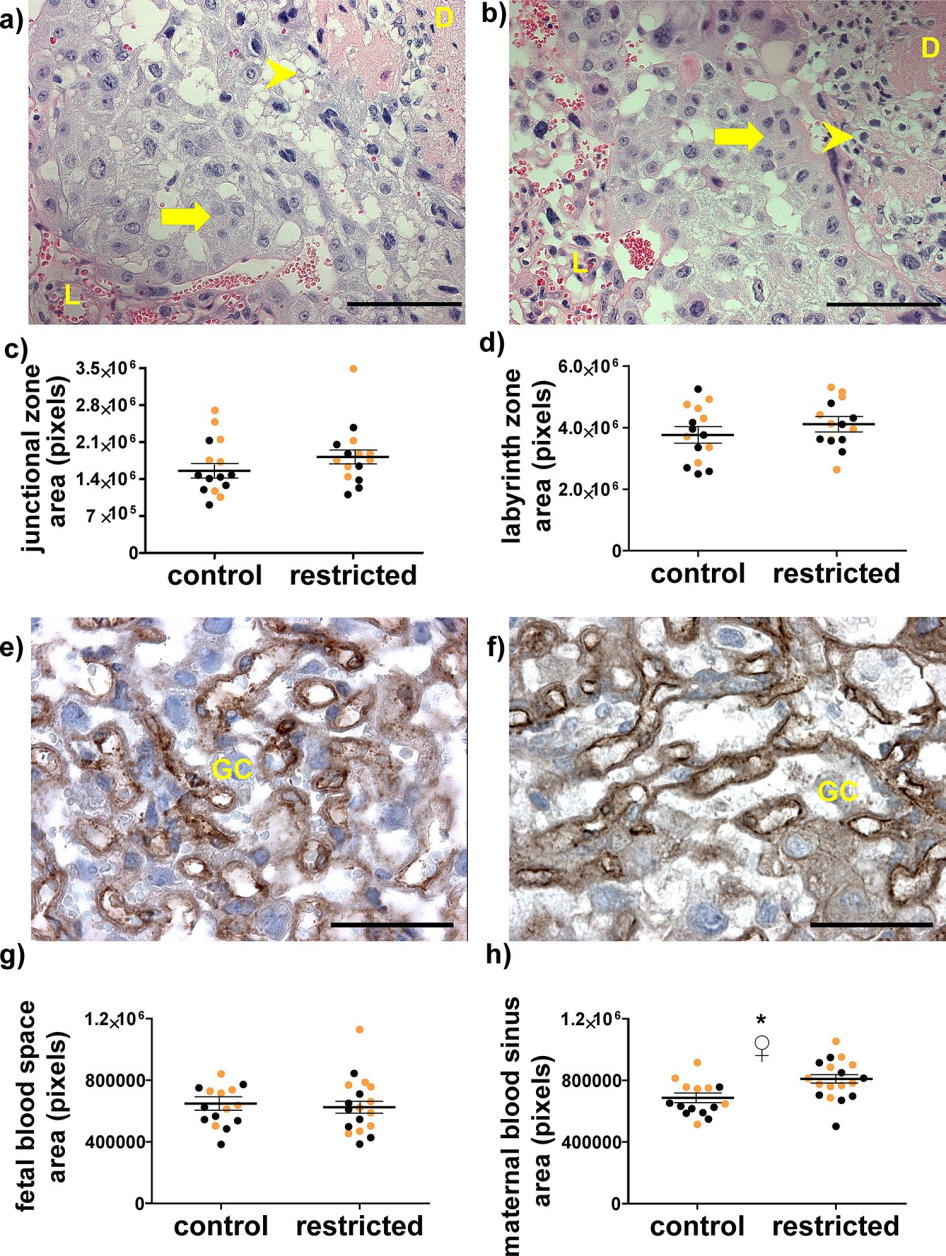
Placental histomorphometry at day 18.5. Representative images of hematoxylin and eosin stained sections of the junctional zone were captured with a 20x objective from control (**a**) and previously food-restricted dams (**b**). L = labyrinth zone, D = decidua, arrow indicates spongiotrophoblast cells, arrowhead indicates glycogen cells. Scale bars are 100 μm. Total cross-sectional areas of the (**c)** junctional and (**d)** labyrinth zone Each marker is one placenta, horizontal lines represent least square means of each maternal treatment group, and error bars are SEM. Representative images of the labyrinth zone were captured with a 40x objective from placentas carried (**e**) control and (**f**) food-restricted dams. Immunoperoxidase staining (brown) indicates endothelial marker PECAM1, showing fetal blood vessels, and nuclei are counterstained with hematoxylin (purple). GC = trophoblast giant cell in maternal sinus. Scale bars are 50 μm. Quantification of (**g**) fetal and (**h**) maternal blood spaces was performed in five sections each from three placentas per dam. Each marker is one placenta, horizontal lines represent least square means of each maternal treatment group, and error bars are SEM.* p <0.05 control vs. restricted. ♀ p<0.05 female vs. male. Black dots are female and gold dots are male.

The overall size of the placenta near term, measured either by weight or cross-sectional area, was not different in dams fed an adequate diet throughout pregnancy and dams restricted during the periconceptional period-mid gestation (**Fig 2A–2D**). No structural abnormalities, hemorrhaging, or necrosis were observed (**Fig 2E and 2F**). Similarly, there were no significant differences in the appearance or cross-sectional areas of the junctional zone (**Fig 3A–3C**). While the total cross-sectional area of the labyrinth zone also was not affected (**Fig 3D**), the structure of blood spaces within it was altered (**Fig 3E–3H**). Specifically, there was an approximately 20% increase in the cross-sectional area of maternal blood sinuses per unit area following periconceptional exposure to food restriction (**Fig 3H**). Maternal blood sinus area also differed by sex, with male placentas having greater area than female placentas, but both sexes responded similarly to food restriction.

### Trophoblast differentiation

**Fig 4:**
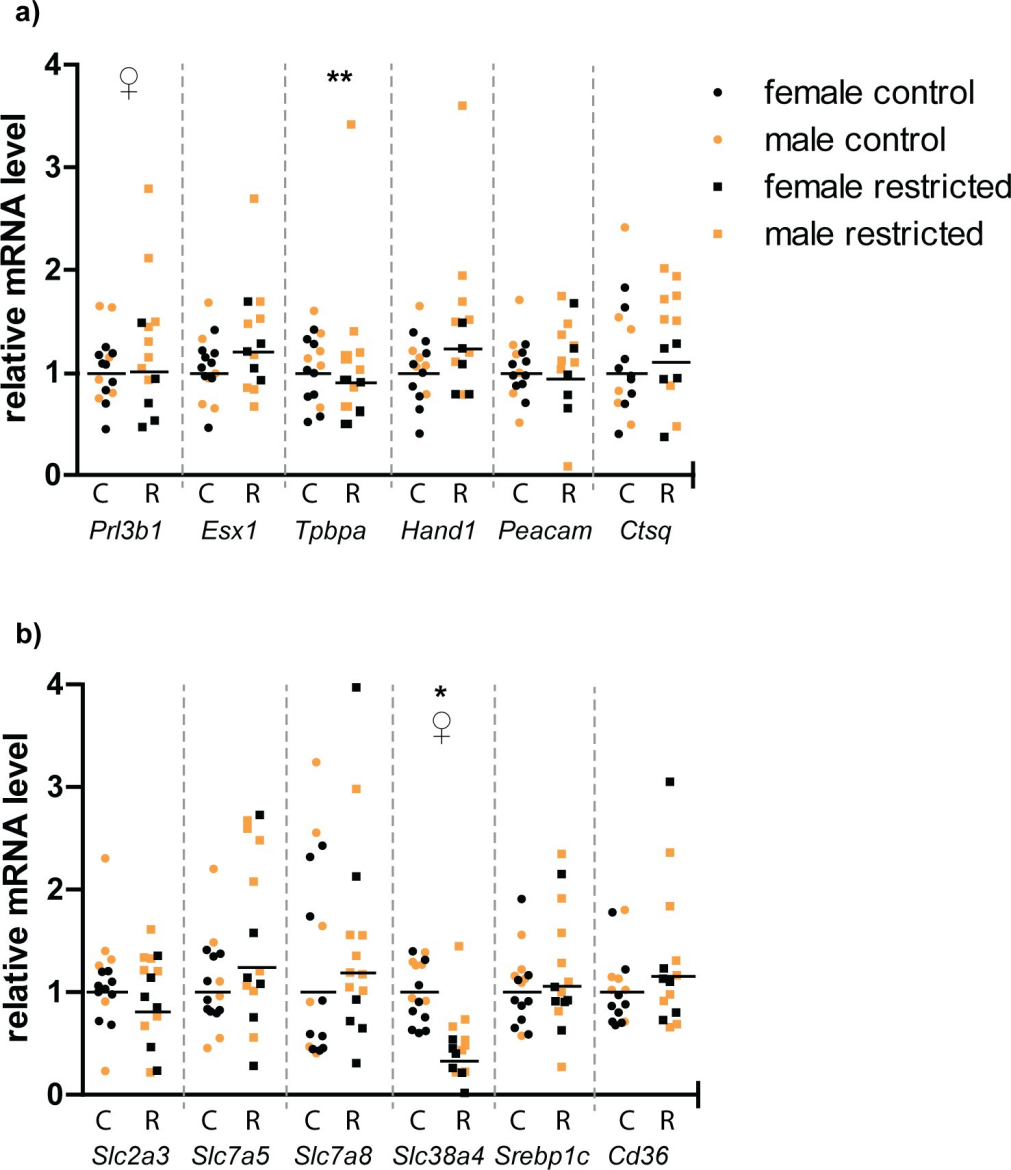
Relative steady-state mRNA levels encoding (a) selected trophoblast lineage markers and (b) key placental nutrient transporters. Relative levels are calculated by the 2^ΔΔCt^ method, using the least square mean of the control group as the reference value, such that the control group = 1. Each symbol represents one placenta. Black horizontal lines represent the 2^ΔΔCt^ for the least square mean of each treatment group. * C = Control, R = restricted. * p<0.05 control vs restricted; ♀ = p<0.05 male vs female; ** = p<0.05 sex x treatment interaction. Placentas were obtained from five dams each in the control and restricted groups.

In order to further characterize the permanent impacts of periconceptional food restriction on placental development, semi-quantitative real-time RT-PCR was used to compare steady-state mRNA levels of transcription factors that are specifically expressed in the major trophoblast sublineages within the placenta (**Fig 4A**). There were no significant differences in relative mRNA levels of placental lineage markers *Esx1* (synctiotrophoblast), *Tpbpa* (spongiotrophoblast and glycogen cells), *Hand1*(trophoblast giant cells), *Prl3b1*(spongiotrophoblast, parietal and sinusoidal trophoblast giant cells) [61], *Ctsq* (sinusoidal trophoblast giant cells) [41] or *Pecam1*(fetal endothelial cells) [37]. However, *Prl3b1* expression did differ with placental sex, with males expressing more than females (p = 0.01). *Tpbpa* expression also was significantly higher in males than in females, but only in the restricted group (p = 0.003) (**Fig 4A**).

### Placental nutrient transporters

Real-time PCR was also used to compare expression of key placental nutrient transporters at d18.5 following periconceptional food restriction (**Fig 4B**). In the placenta from previously food restricted dams, there was a three-fold decrease in mRNA encoding *Slc38a4*, which transports neutral amino acids such as glutamine, glycine, serine and alanine [62]. *Slc38a4* mRNA levels were also higher in males than females, although food restriction had the same effect regardless of sex. In contrast, there were no differences in mRNA levels for the L-type, branched-chain amino acid transporters *Slc7a5* and *Slc7a8* (also known as LAT1 and LAT2). Levels of mRNA for fatty acid transporter *Cd36* and *Srebp1c*, a transcription factor that regulates genes involved in sterol biosynthesis, were not changed, nor were there differences in expression of glucose transporter *Slc2a3* between control and restricted placentae.

### Placental DNA methylation

**Fig 5:**
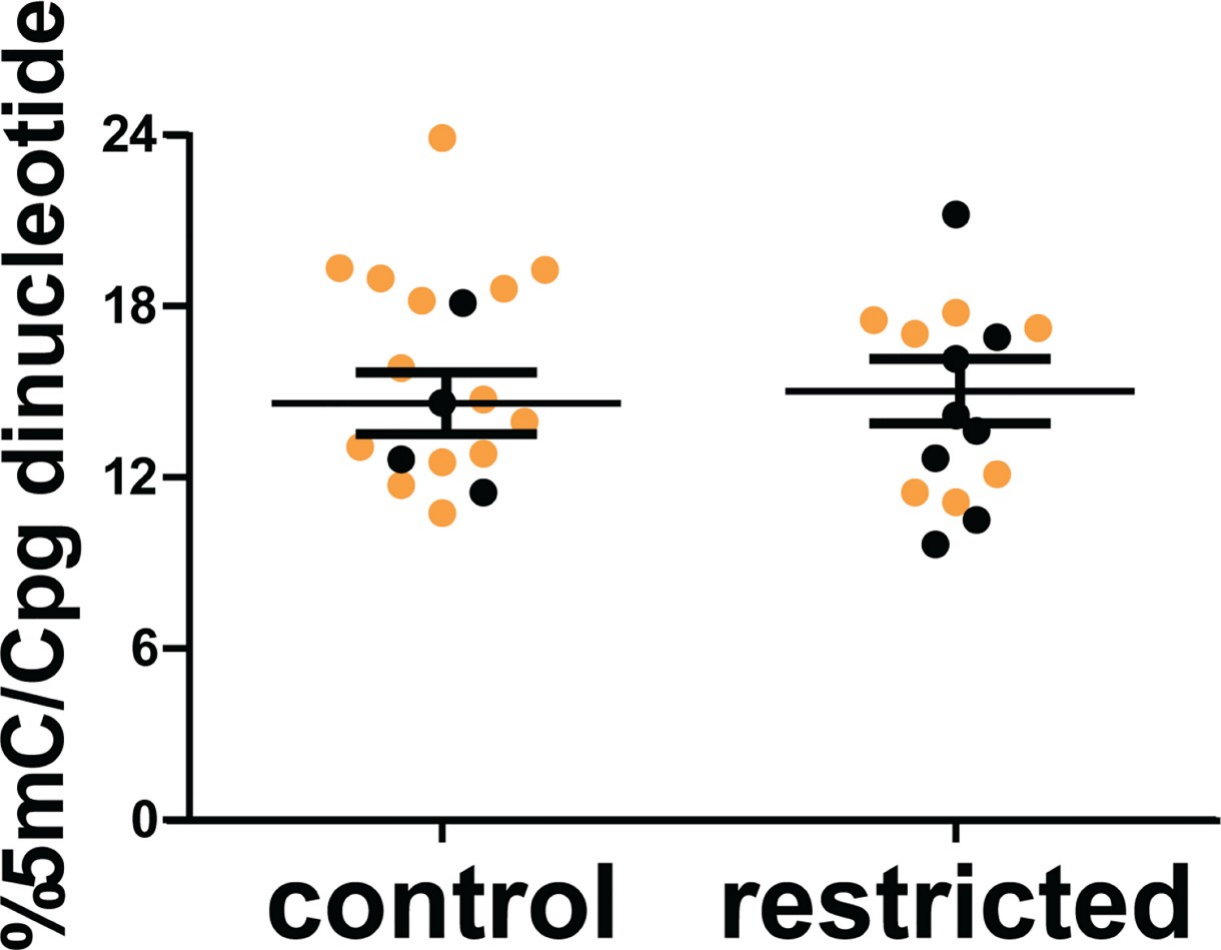
The overall percentage of CpG methylation (%5mC/CpG sites) in placentas from control and periconceptionally food-restricted dams was determined by ELISA. Each marker represents an individual placenta. Black dots are female and gold dots are male. Horizontal lines represent least square means of each maternal treatment group, and error bars are SEM. N = 5 control dams and 6 restricted dams.

An ELISA was used to quantify the effects of periconceptional food restriction on the total level of placental DNA methylation (**Fig 5**). There was no significant difference in the overall percentage of methylated CpGs between placentas from control dams and those exposed to periconceptional undernutrition (p = 0.8), or between male and female placentas (p = 0.4).

## Discussion

The goal of this study was to determine whether the periconceptional period is a critical time in which maternal total caloric restriction can permanently alter placental development and function. Whereas studies in humans and sheep found that total caloric restriction during early pregnancy leads to placental overgrowth near term [20, 22, 23], we previously found that food restriction from days 1.5–11.5 of pregnancy did not change placental characteristics in late pregnancy [21, 22, 63]. In contrast, here we show that food restriction from three weeks prior to mating through d11.5 does alter the structure of the labyrinth zone and the expression of System A neutral amino acid transporter *Slc38a4* (SNAT4), even in late pregnancy.

These results are consistent with the hypothesis that adverse exposures during the periconceptional period, including balanced, total, caloric restriction, have irreversible effects on the placenta. Exposure to a low protein diet from conception to implantation results in a smaller placenta, with greater glucose transport and reduced amino acid transport near term [30, 31]. Alcohol exposure from days -4 to 4 post-conception reduced placental volume and maternal blood space area at day 15 in rats [64], and in sheep, inclusion of serum in the culture medium for the first two days, but not the last two days of preimplantation embryo culture resulted in larger placentas at term [65]. The importance of the periconceptional period has been less clear following total dietary restriction. In “natural” experiments in humans, like the Dutch and Chinese famines, it isn’t possible to distinguish between the periconceptional period and the rest of early pregnancy. In sheep, where this distinction is possible, the periconceptional period appears to be important, but not uniquely important. Restricting ewes to 50% of requirements from days -60 to 30 increased placental vascularity at day 80 [66], although it did not significantly impact placental size or placentome number at day 131 [67]. Nutrient restriction outside the periconceptional period, from days 28–80 of pregnancy was also effective, resulting in a larger placenta, greater placental glucose transporter expression, and greater number of placentomes near term, at day 140–145 (term = 147 days) [22, 68].

The current study, combined with our previous work, shows that in the mouse a period of nutrient restriction that includes periconception (-3 weeks to d11.5) but not the period immediately following (d1.5–11.5), is sufficient for lasting effects on the placenta, strongly suggesting the importance of periconception. However, one limitation to this study is that nutrient restriction was not limited to the periconception period. We cannot rule out the possibility that nutrient restriction both before and after d1.5 are needed. Furthermore, the overall length of the nutrient restriction was longer than in than in the previous study, which could contribute to the more long-lasting effects. Future experiments targeted to just -3 weeks through d1.5, or an even narrower window, would help to more precisely pinpoint the period of susceptibility.

Nonetheless, the window of susceptibility identified in the present study provides some insight into possible mechanisms of action. From -3 weeks to d1.5, the oocyte, and then the zygote, undergo major rewriting of DNA methylation. Maternal imprints are established in the oocyte, and methylation of most non-imprinted genes is erased shortly following fertilization. Evidence from monozygotic twins further shows that many DNA methylation marks are established very early in embryonic development, prior to twinning [69]. A diet deficient in methyl donors (folate, vitamin B12, methionine) during the periconceptional period caused global hypomethylation in fetal sheep in mid-gestation [70]. Caloric restriction throughout gestation is associated with global hypomethylation in mouse placenta at day 19 [71]. This led us to hypothesize that maternal nutrition could alter later placental phenotypes through global DNA methylation differences. However, the overall percentage of methylated CpGs in the d18.5 placentas of control mothers and those that had previously been nutrient restricted were not different. Thus, neither the 50% reduction in macronutrients, nor the accompanying decrease in methyl donors like folate, in the periconceptional period prevented normal overall methylation levels in the late pregnancy placenta. It must be noted that the absence of differences in DNA methylation levels does not rule out differences in individual DNA regions. For example, survivors of the Dutch Hunger Winter show several specific methylation changes in whole blood without differences in global DNA methylation [72]. Additional studies, for example by whole genome bisulfite sequencing, are needed to determine whether any individual differences in placental DNA methylation follow periconceptional undernutrition in the mouse.

Another unknown variable is the effect of refeeding on the growth and function of the placenta. Very little is known about the dynamics of refeeding after nutrient restriction in pregnant mice, and here food consumption was not determined after the resumption of *ad libitum* feeding in the calorie restricted mice. However, it is known that caloric restriction can induce later hyperphagia in non-pregnant mice, and that estrogen plays a role in acute refeeding after fasting [73, 74]. Thus, it is possible that after the food restricted mice were returned to *ad libitum* feeding at day 11.5, that they consumed more calories than the control mice. Additional experiments, including pair-feeding, would be needed to determine whether post-restriction eating plays a role in the placental phenotype on day 18.5.

Placental adaptations in response to maternal undernutrition that outlast the period of undernutrition are potentially harmful because they produce a mismatch between the placenta and the environment. The placenta is “nutrient sensing,” adjusting structure and nutrient transport to preserve fetal growth in the face of reduced maternal nutrient supplies [48]. For example, in the mouse, while the placenta is smaller during maternal food restriction, the size of the labyrinth zone, where nutrients are exchanged, is preserved in comparison to that of the junctional zone [26, 75]. Similarly, regulation of glucose and amino acid transport can compensate for lower nutrient availability, to preserve either maternal or fetal supplies, but if these changes outlast the period of nutrient restriction, they can continue to alter fetal body composition and development. Early pregnancy exposures can alter placental glucose transporter expression later in pregnancy. *Slc2a3* is reduced in the late gestation mouse placenta following assisted reproduction procedures in the periconceptional period [58, 76]. Caloric restriction throughout pregnancy has been shown both to reduce [77] and stimulate [78] placental *Slc2a3* expression in rodents. However, here no significant differences in late gestation gene expression of *Slc2a3* were detected in placentae from mothers that were nutrient restricted during early pregnancy. Along with the previous observation that placental *Slc2a3* levels are acutely regulated by maternal glucose concentrations [79], these results suggest that its expression can change in response to the restoration of food following nutrient restriction.

Amino acid transport is also regulated by maternal nutritional status during pregnancy [80]. The System A neutral amino acid transporter SLC38A4 is a major contributor to fetal growth [47, 81]. Its mRNA, protein and activity levels have been shown to fall in response to various models of nutrient restriction. For example, in rats, a maternal low protein diet throughout gestation reduced its activity [82] and in the baboon, ongoing nutrient restriction reduced microvillous membrane levels of the protein in mid-pregnancy [45]. In contrast, *Slc38a4* mRNA levels increase with periconceptional exposure to assisted reproduction procedures [76]. In the present study, even on d18.5, expression of *Slc38a4* in placentas from previously nutrient restricted dams was only one-third that of controls. This reduction would be predicted to decrease amino acid transport and thereby limit fetal growth. Conversely, placentas from dams that had been nutrient restricted also had increased maternal blood space area within the sampled regions of the labyrinth zone, providing a greater surface area for nutrient exchange between the maternal blood and surrounding syncytiotrophoblast; this would support greater growth. Thus, the overall consequences of the combined placental adaptations for fetal growth and development are difficult to predict, but the observed lack of change in fetal weights could be a result of these placental alterations canceling one another. While both alterations were present at d18.5, it is possible that one occurred first, triggering a compensatory response to maintain consistent amino acid transport.

Offspring exposed to undernutrition during early pregnancy have greater chronic disease risk as adults, even without changes in birth weight [10]. Our data support the idea that maternal food deprivation during the periconceptional period is particularly important for placental function throughout pregnancy even if nutrition is later restored [83–85]. Additionally, the findings suggest that careful examination of the term placenta may provide evidence of adverse exposures in the periconception period, perhaps even serving as an indicator of future health.
